# First Report of Nephrocalcinosis in Aquacultured Brazilian Sardine (*Sardinella brasiliensis* Steindachner, 1879)

**DOI:** 10.1111/jfd.70081

**Published:** 2025-10-24

**Authors:** Danielle Souza Vieira, Caio Francisco Santana Farias, Marco Shizuo Owatari, Mauricio Laterça Martins, Aline Brum, Caio Magnotti

**Affiliations:** ^1^ LAPMAR—Marine Fish Farming Laboratory, Department of Aquaculture, School of Agricultural Sciences (CCA) Federal University of Santa Catarina (UFSC) Florianópolis Santa Catarina Brazil; ^2^ AQUOS—Aquatic Organisms Health Laboratory, Department of Aquaculture, School of Agricultural Sciences (CCA) Federal University of Santa Catarina (UFSC) Florianópolis Santa Catarina Brazil

**Keywords:** aquaculture, fish health, nephrolithiasis, pathology

## Abstract

While nephrocalcinosis (kidney stones) is uncommon in wild teleost fish, various environmental and nutritional factors could lead to its occurrence in aquacultured fish. This study presents the first documented case of kidney stones in aquacultured Brazilian sardine ( *Sardinella brasiliensis* ). During necropsy, eighteen hard, white kidney stones were found in the posterior kidney, with an average diameter of 3.71 mm and a total length of 16.8 mm. Morphological analysis revealed stones of different sizes and shapes, including elongated and irregular structures. This discovery enhances our understanding of pathological conditions in *S. brasiliensis* and underscores the importance of further research into the causes, prevalence and potential implications for fish health and fisheries sustainability.

## 1 Introduction

The Brazilian sardine ( *Sardinella brasiliensis* ) is a crucial fisheries resource in Brazil, facing threats from overfishing. Aquaculture has emerged as a key solution for mass production of this species, whether for stock replenishment or direct use in the canning industry (Owatari et al. 2024). Recent studies have shown the technical feasibility of stock formation and reproduction (Magnotti et al. 2020), nutrition (Baloi et al. 2016) and technological advancements (Owatari et al. 2023) to support the cultivation of Brazilian sardine in Brazilian marine fish farming. Nevertheless, creating an optimal cultivation environment and developing species‐specific artificial feeds remain challenges in marine fish farming. In aquaculture, fish pathologies are frequently linked to farming systems and nutritional factors (Islam et al. 2024). As a result, there is still a substantial gap in scientific knowledge regarding the pathologies affecting the health of Brazilian sardine.

Nephrocalcinosis (kidney stones) in fish (Klykken et al. 2022; Minarova et al. 2023; Takvam et al. 2023) is a disorder that shares similarities with kidney stone formation in other vertebrates. It mainly involves urinary supersaturation and the crystallisation of minerals like calcium oxalate (Khoo 2019; Wang et al. 2021; Chen et al. 2023). Although the prevalence of nephrocalcinosis in wild fish is unknown (Takvam et al. 2023), several environmental and nutritional factors may lead to its occurrence in aquacultured fish (Klykken et al. 2022; Minarova et al. 2023). The risk factors, prevention strategies and consequences in aquacultured fish like Brazilian sardine are not yet fully understood.

Environmental factors, water quality changes, inadequate diets and exposure to infectious agents can increase the risk of nephrocalcinosis in fish, leading to metabolic disturbances and inflammation that promote crystal deposition in the kidneys (Khoo 2019; Klykken et al. 2022; Minarova et al. 2023). The presence of kidney stones can impair vital physiological functions, directly impacting fish survival and overall health (Klykken et al. 2022), underscoring the importance of environmental and nutritional management to prevent this condition.

Recently, during a regular haematological assessment at the Marine Fish Farming Laboratory (LAPMAR), it was discovered that sardines had decreased haematocrit levels, indicating a potential case of anaemia. Subsequent investigation uncovered kidney stones in the Brazilian sardine population.

To date, there have been no reports of nephrocalcinosis in aquacultured Brazilian sardine. This study presents the first documented case of nephrocalcinosis in *S. brasiliensis* and discusses the potential causes of kidney stone development and the health implications for affected fish.

## 2 Materials and Methods

The diagnosis of nephrocalcinosis was performed on fish farmed at the LAPMAR of the Federal University of Santa Catarina (UFSC), Barra da Lagoa, Florianópolis, SC, Brazil. The fish were fed a commercial diet (45% crude protein) and housed in a circular PVC fish farm pond measuring 1.0 m in height and with a capacity of 10,000 L. The pond was connected to a recirculating aquaculture system (RAS) with physical and biological filtration, constant temperature and aeration. The pond water quality had a salinity of 33.0 ± 0.9 ppt, temperature of 18.6°C ± 0.5°C, dissolved oxygen of 5.39 ± 0.3 mg L^−1^, pH of 7.94 ± 0.10, total ammonia of 0.2 ± 0.01 mg L^−1^, nitrite of 0.27 ± 0.03 mg L^−1^ and alkalinity of ≥ 100 mg CaCO_3_ L^−1^.

LAPMAR periodically assesses the health of its fish stocks. For this, all fish were anaesthetised with 50 mg L^−1^ of benzocaine (Sterzelecki et al. 2018), measure and weighed. A routine haematological evaluation was conducted on 22 Brazilian sardines with an average weight of 53.00 ± 3.01 g. Results showed reduced haematocrit levels in 16 individuals (average of 15.6% ± 8.16%), suggesting a possible case of anaemia.

Subsequently, the fish were euthanised using a deep anaesthetic procedure with 200 mg L^−1^ of benzocaine (Scheuer et al. 2024), and necropsies were then performed on all 22 Brazilian sardine specimens to conduct a thorough examination of their internal organs. During the renal inspection, kidney stones (nephrocalcinosis) of various sizes and shapes were grossly discovered in 12 of the 16 fish with decreased haematocrit levels. The stones were removed and examined using a ZOOM DI‐152 T trinocular stereoscopic microscope and were measured with Opto‐Edu Image View software. Samples of nearby renal tissue were preserved in 10% buffered formalin for future histopathological and stone composition analyses.

## 3 Results

**FIGURE 1:**
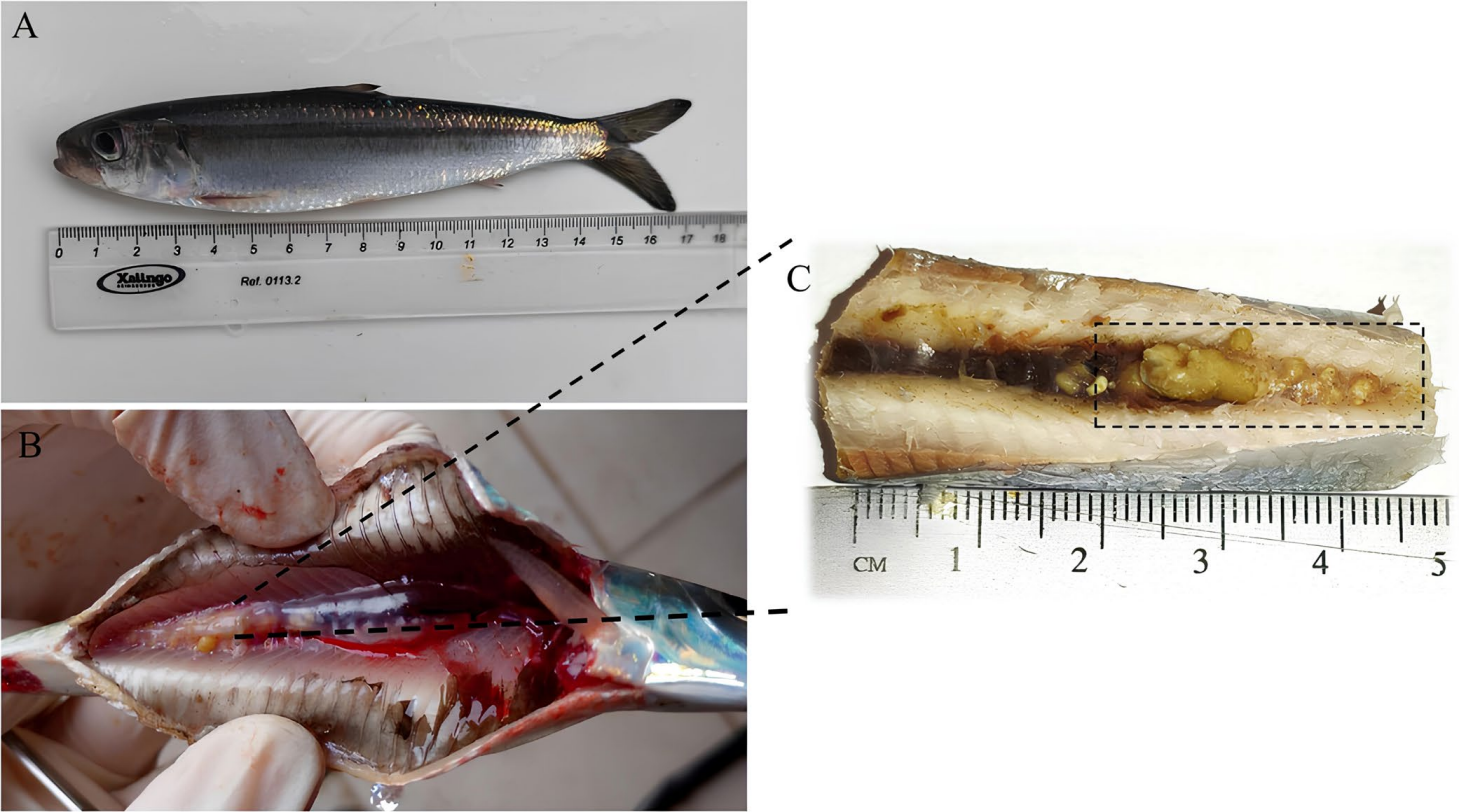
In (A), a specimen of a Brazilian sardine ( *Sardinella brasiliensis* ) diagnosed with nephrocalcinosis (kidney stone). In (B, C), nephrocalcinosis is observed in the posterior kidney of the Brazilian sardine with multiple stones of varying sizes.

**FIGURE 2:**
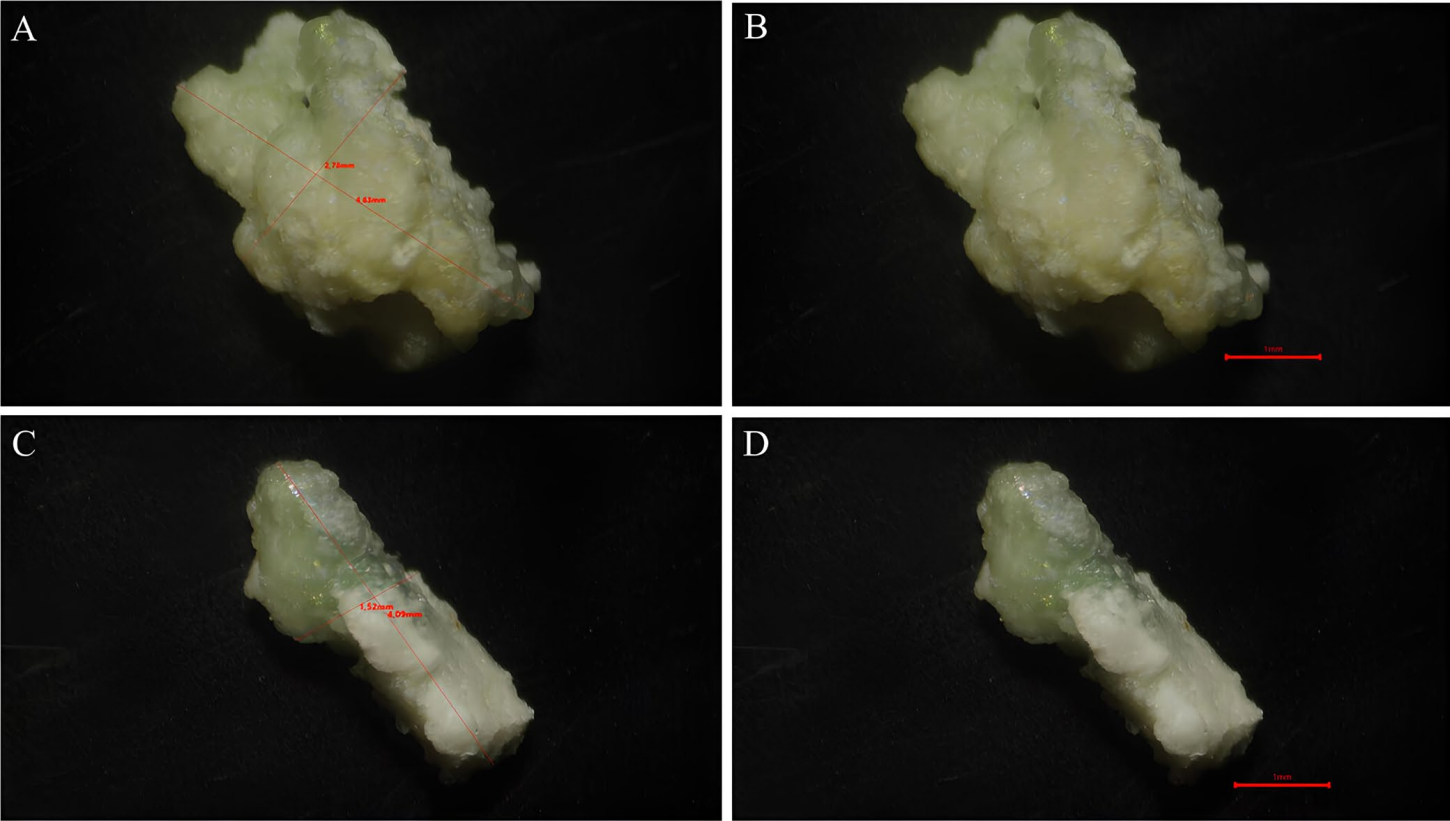
Kidney stone removed from the posterior kidney of the Brazilian sardine ( *Sardinella brasiliensis* ). (A and B) Irregularly shaped stone measuring approximately 4.63 mm × 2.78 mm. (C and D) Elongated stone measuring approximately 4.09 mm × 1.52 mm. Scale bar: 1 mm.

The Brazilian sardines that were analysed had an average total length of 17.0 ± 1.0 cm, with 12 of them having kidney stones (Figure 1A). The kidney stones were mainly found in the posterior kidney, with an average length of 16.80 mm. Various stones of different sizes and shapes were present in the posterior kidney, including isolated formations of around 1.0 cm and others ranging from 0.1 to 0.4 mm (Figure 1B,C). During the necropsy, a total of eighteen kidney stones were observed, with colours ranging from whitish to yellowish and a very rigid consistency (Figure 2). The dimensions varied, with an average diameter of 3.71 mm, a length of 4.36 mm and a width of 2.15 mm. Morphologically, the kidney stones showed distinct patterns, with some being irregular (4.63 × 2.78 mm) (Figure 2A) and others elongated (4.09 × 1.52 mm) (Figure 2C).

## 4 Discussion

This is the first report of nephrocalcinosis, popularly known as kidney stones, in aquacultured Brazilian sardines ( *S. brasiliensis* ). Nevertheless, previous research has documented nephrocalcinosis in aquacultured fish. Klosterhoff et al. (2015) reported kidney stones ranging from 4.0 to 13 mm in diameter in Cobia ( *Rachycentron canadum* ), which are larger than those found in Brazilian sardines in this study. However, these stones were discovered in fish weighing over 4.0 kg, which are considerably larger.

This severe pathophysiology was suspected during a regular haematological assessment. Haematological tests can uncover underlying physiological issues like infections or organ damage (Fazio 2019). Anaemia can be an early complication of chronic renal failure (Kazmi et al. 2001), underscoring the importance of hematologic analyses prior to diagnosing kidney stones. Haematological tests in fish can serve as a health status indicator, detecting physiological imbalances even before obvious clinical signs appear (Owatari et al. 2020). In this study, a basic routine haematological examination proved to be a crucial tool in evaluating the health of sardines and offering valuable insights into their physiological and pathological conditions. Blood analysis helped identify changes indicating a potential metabolic/nutritional disorder or an environmental issue linked to captivity.

Environmental factors may also play a role in the development of nephrolithiasis in fish like *S. brasiliensis* . The kidney is essential for regulating acid–base balance and excreting ammonia in freshwater and marine fish (Takvam et al. 2023). Understanding the relationship between water quality and fish health is crucial (Minarova et al. 2023), especially in relation to the development of nephrocalcinosis. Despite pond water quality parameters being within acceptable ranges for all tested variables in this study, nephrocalcinosis still occurred in the fish. Therefore, it is essential to explore potential contributing factors like gas supersaturation or elevated levels of dissolved gases, which are common in unbalanced recirculating systems. Additionally, increased temporary hardness or excessive alkalinity can encourage calcium deposition in soft tissues, emphasising the need for further investigation (Takvam et al. 2023).

The development of nephrocalcinosis may be influenced by various factors, such as aquatic environment hyperoxia and hypercapnia, leading to an increase in blood carbon dioxide levels and disturbances in blood acid–base balance. This can initiate a metabolic compensation process that ultimately results in the formation and accumulation of calcium phosphate in tissues (Minarova et al. 2023). One hypothesis we considered regarding the formation of kidney stones is linked to nutritional factors. In Brazil, there are no companies that produce specialised feed for marine fish, which could result in nutritional gaps in the fish. From a nutritional perspective, it is understood that diets high in animal protein, low in calcium, high in sodium and low in water intake may elevate the likelihood of kidney stone development in animals (Siener 2021). Elements like an excessively mineral‐rich diet may pose potential risks for marine fish (Lall and Kaushik 2021), although the physiology of these fish provides protective mechanisms against nephrolithiasis formation (Whittamore 2020; Siener 2021).

The exact causes of nephrocalcinosis in teleosts are not fully understood, but it is believed that acid–base imbalances play a significant role in its development (Minarova et al. 2023). Factors such as strenuous movements and environmental hypoxia can disrupt the acid–base balance in fish. This disruption leads to the production of acid equivalents from the breakdown of adenylate and the generation of lactate, resulting in metabolic acidosis caused by the accumulation of CO_2_ (respiratory acidosis). High ambient partial pressure of carbon dioxide (PCO_2_) can also contribute to respiratory acidosis, especially in closed‐system aquaculture settings (Takvam et al. 2023). These conditions are especially pertinent for Brazilian sardines, a pelagic species known for their continuous active swimming in aquaculture ponds. Additionally, the fish in the study were reared in closed‐system RAS, which may account for the observed pathophysiology. This study presents the initial documentation of nephrocalcinosis in *S. brasiliensis* , enhancing our understanding of the pathology in this species. This discovery underscores the importance of conducting additional research on the causes and prevalence of this condition, as well as its implications for fish health in aquaculture and potential strategies for preventing the formation of kidney stones.

## Author Contributions

Danielle Souza Vieira: investigation, methodology, formal analysis. Caio Francisco Santana Farias: investigation, methodology, formal analysis. Marco Shizuo Owatari, Mauricio Laterça Martins, Aline Brum, and Caio Magnotti: project administration, funding, writing – original draft, writing – review and editing.

## Ethics Statement

All the procedures were authorised by the Animal Ethics Committee (CEUA No. 3741180425).

## Conflicts of Interest

The authors declare no conflicts of interest.

## Data Availability

The data that support the findings of this study are available from the corresponding author upon reasonable request.

## References

[jfd70081-bib-0001] Baloi, M. , C. V. Carvalho , F. C. Sterzelecki , G. Passini , and V. R. Cerqueira . 2016. “Effects of Feeding Frequency on Growth, Feed Efficiency and Body Composition of Juveniles Brazilian Sardine, *Sardinella Brasiliensis* (Steindacher 1879).” Aquaculture Research 47, no. 2: 554–560. 10.1111/are.12514.

[jfd70081-bib-0002] Chen, T. , B. Qian , J. Zou , et al. 2023. “Oxalate as a Potent Promoter of Kidney Stone Formation.” Frontiers in Medicine 10: 1159616. 10.3389/fmed.2023.1159616. 37342493 PMC10278359

[jfd70081-bib-0003] Fazio, F. 2019. “Fish Hematology Analysis as an Important Tool of Aquaculture: A Review.” Aquaculture 500: 237–242. 10.1016/j.aquaculture.2018.10.030.

[jfd70081-bib-0004] Islam, S. I. , F. Ahammad , and H. Mohammed . 2024. “Cutting‐Edge Technologies for Detecting and Controlling Fish Diseases: Current Status, Outlook, and Challenges.” Journal of the World Aquaculture Society 55, no. 2: e13051. 10.1111/jwas.13051.

[jfd70081-bib-0005] Kazmi, W. H. , A. T. Kausz , S. Khan , et al. 2001. “Anemia: An Early Complication of Chronic Renal Insufficiency.” American Journal of Kidney Diseases 38, no. 4: 803–812. 10.1053/ajkd.2001.27699. 11576884

[jfd70081-bib-0006] Khoo, L. 2019. “Renal Diseases and Disorders.” In Fish Diseases and Medicine, 1st ed., 211–229. CRC Press. 10.1201/9780429195259.

[jfd70081-bib-0007] Klosterhoff, M. , V. Pedrosa , L. A. Sampaio , L. Ramos , M. B. Tesser , and L. A. Romano . 2015. “Nephrocalcinosis and Kidney Stones in *Rachycentron Canadum* .” Bulletin of the European Association of Fish Pathologists 35, no. 4: 139.

[jfd70081-bib-0008] Klykken, C. , A. K. Reed , A. S. Dalum , et al. 2022. “Physiological Changes Observed in Farmed Atlantic Salmon ( *Salmo salar* L.) With Nephrocalcinosis.” Aquaculture 554: 738104. 10.1016/j.aquaculture.2022.738104.

[jfd70081-bib-0009] Lall, S. P. , and S. J. Kaushik . 2021. “Nutrition and Metabolism of Minerals in Fish.” Animals 11: 2711. 10.3390/ani11092711. 34573676 PMC8466162

[jfd70081-bib-0010] Magnotti, C. , F. C. Sterzelecki , F. Cipriano , et al. 2020. “Spontaneous Spawning of Brazilian Sardine in Captivity.” Boletim do Instituto de Pesca 46, no. 1: e539. 10.20950/1678-2305.2020.46.1.539.

[jfd70081-bib-0011] Minarova, H. , M. Palikova , R. Kopp , et al. 2023. “Nephrocalcinosis in Farmed Salmonids: Diagnostic Challenges Associated With Low Performance and Sporadic Mortality.” Frontiers in Veterinary Science 10: 1121296. 10.3389/fvets.2023.1121296. 37152688 PMC10157097

[jfd70081-bib-0012] Owatari, M. S. , V. R. Cerqueira , M. F. Baloi , et al. 2024. “Low‐Trophic‐Level Species *Sardinella Brasiliensis* in Aquaculture: Crosstalk Between Aquaculture and Fisheries.” Reviews in Fisheries Science & Aquaculture 32, no. 4: 679–697. 10.1080/23308249.2024.2362236.

[jfd70081-bib-0013] Owatari, M. S. , G. F. A. Jesus , L. Cardoso , N. B. Lehmann , M. L. Martins , and J. L. P. Mouriño . 2020. “Can Histology and Haematology Explain Inapparent *Streptococcus agalactiae* Infections and Asymptomatic Mortalities on Nile Tilapia Farms?” Research in Veterinary Science 129: 13–20. 10.1016/j.rvsc.2019.12.018. 31901532

[jfd70081-bib-0014] Owatari, M. S. , C. Magnotti , J. H. Vargas , C. V. A. Carvalho , F. C. Sterzelecki , and V. R. Cerqueira . 2023. “Influence of Salinity on Growth and Survival of Juvenile *Sardinella Brasiliensis* .” Boletim do Instituto de Pesca 49: e808. 10.20950/1678-2305/bip.2023.49.e808.

[jfd70081-bib-0015] Scheuer, F. , M. S. Owatari , E. M. Brasil , et al. 2024. “Dietary Enrichment With Fish Oil Improved n–3 LC‐PUFA Profile in Aquacultured *Sardinella Brasiliensis* Fillet.” Journal of Food Composition and Analysis 127: 105978. 10.1016/j.jfca.2024.105978.

[jfd70081-bib-0016] Siener, R. 2021. “Nutrition and Kidney Stone Disease.” Nutrients 13, no. 6: 1917. 10.3390/nu13061917. 34204863 PMC8229448

[jfd70081-bib-0017] Sterzelecki, F. C. , J. K. Sugai , M. Baloi , et al. 2018. “Effects of Increasing Protein Level on the Performance, Enzyme Activity and Body Composition of the Brazilian Sardine, *Sardinella brasiliensis* (Steindachner, 1879).” Aquaculture Nutrition 24, no. 1: 366–374. 10.1111/anu.12567.

[jfd70081-bib-0018] Takvam, M. , C. M. Wood , H. Kryvi , and T. O. Nilsen . 2023. “Role of the Kidneys in Acid‐Base Regulation and Ammonia Excretion in Freshwater and Seawater Fish: Implications for Nephrocalcinosis.” Frontiers in Physiology 14: 1226068. 10.3389/fphys.2023.1226068. 37457024 PMC10339814

[jfd70081-bib-0019] Wang, Z. , Y. Zhang , J. Zhang , Q. Deng , and H. Liang . 2021. “Recent Advances on the Mechanisms of Kidney Stone Formation.” International Journal of Molecular Medicine 48, no. 2: 149. 10.3892/ijmm.2021.4982. 34132361 PMC8208620

[jfd70081-bib-0020] Whittamore, J. M. 2020. “The Teleost Fish Intestine Is a Major Oxalate‐Secreting Epithelium.” Journal of Experimental Biology 223, no. 12: jeb216895. 10.1242/jeb.216895. 32122927

